# Concurrent validity of the non-exercise based VO_2_max prediction equation using percentage body fat as a variable in asian Indian adults

**DOI:** 10.1186/1758-2555-4-34

**Published:** 2012-09-21

**Authors:** Shweta Shenoy, Bhupinder S Tyagi, Jaspal S Sandhu

**Affiliations:** 1 Reader, Department of Sports Medicine & Physiotherapy, Guru Nanak Dev University, Amritsar, Punjab, India

## Abstract

**Background:**

Aerobic capacity (VO_2_max) is highly dependent upon body composition of an individual and body composition varies with ethnicity. The purpose of this study was to check the concurrent validity of the non-exercise prediction equation developed by Jackson and colleagues (1990) using percentage body fat as a variable in Asian Indian adults.

**Methods:**

One hundred twenty college-aged participants (60 male, 60 female, mean age 22.02 ± 2.29 yrs) successfully completed a maximal graded exercise test (GXT) on a motorized treadmill to assess VO_2_max. VO_2_max was then estimated by the non-exercise prediction equation developed by Jackson and colleagues (1990) using percentage body fat. Percentage body fat was calculated by three different models (Sandhu et al’s fat mass equation, Durnin-womersley’s 4 site percentage body fat and Jackson & Pollock’s 4 site percentage body fat) and was used in the above equation. The results of VO_2_max obtained using “gold standard” treadmill methods were then compared with the three results of VO_2_max obtained by Jackson et al’s equation (using three different models to calculate percentage body fat) and it was determined which equation is best suited to determine percentage body fat and in turn VO_2_ max for Indian population.

**Results:**

Jackson et al’s prediction equation overpredicts VO_2_max in Asian Indian subjects who have a lower VO_2_max (33.41 ± 14.39 ml/kg/min) than those reported in other age matched populations. percentage body fats calculated by the three equations were significantly different and the correlation coefficient (r) between VO_2_max calculated by Jackson and colleagues (1990) using Sandhu et al’s equation for percentage body fat with VO_2_ max calculated using treadmill (gold standard) (r = .817) was found slightly more significantly correlated than the other two equations and was not statistically different from the measured value.

**Conclusions:**

This study proves that VO_2_max equation using Sandhu et al’s model for percentage body fat yields more accurate results than other studied equations in healthy college-aged participants in India.

## Background

Maximal oxygen uptake or consumption (VO_2_max) is the maximum capacity of an individual’s body to transport and use oxygen during incremental exercise which reflects the physical fitness of an individual [1]. The measurement of VO_2_max as a parameter of cardio-respiratory fitness is useful when educating individuals about their overall fitness status, considering cardiovascular risk such as physical inactivity and overweight status [2]. Since this method involves the direct measurement of expired gas samples, it is considered highly reliable and accurate and is, thus considered the “gold standard” for determining VO_2_ max [3]. This approach though is practically difficult, requires fairly expensive equipment and the assistance of trained personnel. Accurately measuring the VO_2_max involves a physical effort sufficient in duration and intensity to fully tax the aerobic system, the performance of which is influenced by the individual’s motivation. This intense effort makes the procedure difficult to perform because of which, some older or higher-risk individuals should not perform the test without medical supervision **[**[4].

As a result, researchers have generated various non- exercise based tests for predicting VO_2_max that utilize variables of physical parameters (viz. age, gender, height, weight, BMI, body fat percentage) [4]. There are very few valid models available which utilize percentage body fat as a determinant of VO_2_max.These models include those of Heil *et al.* (1995) [5] and Jackson *et al.* (1990) [6]. Body composition is known to vary with ethnicity [7]. People of South Asian origin have a higher percentage of body fat for any given BMI while those of Black Caribbean origin have a lower percentage body fat for any given BMI [8-11], (c.f. Higgins,2008) [12]. Higher amounts of body fat and obesity are associated with increased risk of adverse health events and greater mortality [13]. Obesity has been defined by the American College of Sports Medicine [2] as an excessive amount of adipose tissue, which is defined in young adults as body fat > 25percentage in males and >32percentage in females.

Percentage of body fat is usually assessed by using skinfold thickness in clinical & field setting because this method is simple to use and low in cost [14]. Regression equations are used to calculate body composition (body density, percentage body fat) from these skinfold measurements [15]. However, as all these prediction equations are based on mathematical relationships, they predict body composition only if population resembles the reference population that was used in the development of the prediction equation [16]. Most of the existing equations or the estimation of body fat percentage for example Jackson and Pollock [6] and Durnin and Womersley [17] which are widely used in epidemiological studies using skinfolds have been developed and validated in the Caucasian population and tend to under predict body fat percentage in Asian Indians. Considering the ethnic differences in fat percentage in 2010, Sandhu *et al*. [18] developed a body fat mass prediction equation for young Indian adults using skinfold data and body fat percentage using under water weighing.

Several non exercise prediction equations for the estimation of VO_2_max use percentage body fat in their formulation. Again these equations were also developed and validated in ethnic populations other than Indians. The ethnic differences in percentage body fat may make them unsuitable for use in Asian Indians. Jackson *et al.* (1990) [6] & Heil *et al.*(1995) [5] proposed prediction models which use body fat percentage of an individual in their formulation. These equations have been shown to be fairly reliable and valid. Researchers [19] have checked the cross-validation of non-exercise models (N-Ex) [6] and have found it accurate for healthy adults and suggested its use in epidemiological studies.

We believe that these N-Ex prediction equations may not be very accurate in the Asian Indian population because of the inherent differences in body compositions that exist in different races, specifically the fact that for a given BMI the percentage body fat in Asian Indians is higher [20] and hence a limited sub maximal aerobic capacity and reduced VO_2_max relative to body weight [21] Jackson [6] equation has been widely used in epidemiological studies. Since it utilizes body fat percentage in its formulation, our study was designed with the objective of trying to examine the correlation between lab measurement of VO_2_max and the VO_2_max calculated through [6] NEX prediction equation. As it incorporates an individual’s body fat percentage we decided to use the body fat percentage calculated through 3 methods and analyse which of these predicted VO_2_max values more closely to the lab measured VO_2_max. Lastly we wanted to analyze the validity of this non-exercise prediction equation using body fat percentage calculated through the above methods.

## Methods

### Participants and procedures

One hundred twenty college students (males = 60; females = 60), aged 18 to 27 year, satisfied the requirements of this study. Volunteers, recruited for the study, were primarily the students of Department of Sports Medicine & Physiotherapy, Guru Nanak Dev University, Amritsar, Punjab India. Criteria for inclusion in the study were, participants who were non athletic, but who occasionally participated in recreational activities, who were able to perform exercise, with no prior history of cardiovascular, peripheral vascular, respiratory diseases, malignancy, orthopaedic or musculoskeletal lesion. Subjects should have been non smokers, non alcoholic, not taking any medications and non diabetics. All participants signed an informed consent document in accordance with University ethical Committee guidelines.

### Non exercise questionnaires and physical measurements

Prior to testing, all subjects completed

*brief Physical Activity Readiness Questionnaire (PAR-Q) [22] to screen for cardiovascular contraindications.

*Physical Activity Recall (PA-R) questionnaire (0–7 scale) **[**[6].

### The physical activity rating (PAR) was determined as follows

The participant was asked to give the appropriate PAR score (0–7) based on the following scale:

I. Does not participate regularly in programmed recreation, sport, or physical activity.

0 points: Avoids walking or exercise (for example, always uses elevators, drives whenever possible instead of walking).

1 point: Walks for pleasure, routinely uses stairs, occasionally exercises sufficiently to cause heavy breathing or perspiration.

II. Participates regularly in recreation or work requiring modest physical activity (such as golf, horseback riding, calisthenics, gymnastics, table tennis, bowling, weight lifting, or yard work).

2 points: 10–60 min per week

3 points: Over 1 h per week

III. Participates regularly in heavy physical exercise (such as running or jogging, swimming, cycling, rowing, skipping rope, running in place) or engages in vigorous aerobic type activity (such as tennis, basketball, or handball).

4 points: Runs less than 1 mile per week or spends less than 30 min per week in comparable physical activity.

5 points: Runs 1–5 miles per week or spends 30–60 min per week in comparable physical activity.

6 points: Runs 5–10 miles per week or spends 1–3 h per week in comparable physical activity.

7 points: Runs more than 10 miles per week or spends more than 3 h per week in comparable physical activity.

Four-site skinfold measures were evaluated for females and males (biceps, triceps, subscapularis, and suprailiac) using Harpenden caliper to determine percent body fat (percentage fat).

Participants were instructed to drink plenty of water and abstain from strenuous exercise for 24 h prior to testing, and not to consume food, alcohol, caffeine, or tobacco products three hours before testing [2]. Each participant was fitted with a HR monitor (Polar Inc., NY) to measure exercise HR during the maximal GXT on a calibrated motor driven treadmill (Vacuumed Vista MX, California, USA). Participants’ height and body mass were measured and recorded while wearing lightweight clothing and no shoes, using a stadiometer and calibrated digital weight scale(model:EB-EQ90,equinox overseas (P) Ltd., India). Instructions about the maximal GXT (involving the protocol, electronic heart rate monitor, mouth piece, and rating of perceived exertion (RPE) scale [23] were given to all participants prior to testing. Also participants were allowed to practice treadmill walking/jogging as needed to become familiar with the equipment.

### Maximal treadmill graded exercise test

Participants performed a maximal GXT using Bruce graded GXT protocol [24] consists of seven stages. Each stage comprised of 3 mins. During the maximal GXT, metabolic gases were collected using the Vacuumed Vista MX metabolic measurement system (California, USA). The VO_2_max and respiratory exchange ratio (RER) were computed, averaged, and printed by an online computer system every 10 seconds. The participants’ exercise heart rate (HR) and RPE score was recorded at the end of each stage. VO_2_max was considered valid when at least two of the following criteria were met [4]:

1 Maximal heart rate within 15 beats of age predicted maximal heart rate [25].

2 Respiratory exchange ratio equal to or greater than 1.10 [26].

3 RPE equal to or greater than 17.

4 The subject did not show any increase in oxygen consumption even after increasing the intensity of exercise.

5 Participants who failed to meet at least two of these criteria were dropped from the study.

Body fat percentage was then calculated by using three different formulae i.e.

1 Sandhu et al’s fat mass equation [18] (S’s eqn)

2 Durnin-Womersley’s 4 site percentage body fat [17] (D-W ‘s eqn)and

3 Jackson & Pollock’s 4 site percentage body fat [27]. (J-Ps eqn)

The calculated body fat percentage was utilized in the estimation of VO2max by using **Jackson et al’**s [6] prediction equation i.e.

(1) $$\begin{array}{l} N-\text{Ex}\;VO_{2}\text{max}\;\text{using}\;\text{percentageF}\\ \;=50.513+1.589\;\left(\text{History}\;\text{of}\;\text{physical}\;\text{fitness}\;0–7\right)\\ \;–\;0.289\;\left(\text{age}\right)–0.552\;\left(\text{percentageF}\right)\\ \;+5.863\;\left(\text{gender}\;0–1\right)\text{.} \end{array}$$

#### Statistics

Three different formulae for the calculation of body fat percentage for males and females were applied and thus three different values of the same for both males and females were obtained. ANOVA was applied to test for the statistical significance between these values followed by Scheffe’s post hoc test. Then VO_2_max was calculated by substituting these body fat percentages in Jackson et al’s (1990) [6] equation. Again ANOVA was applied to the VO_2_max calculated through different percentage body fat followed by post hoc Scheffe’s test. Inter- class correlation matrix was also used on the calculated VO_2_ max (through substituting percentage body fats) to test the correlation between the measured VO_2_max and the calculated VO_2_max.

## Results

Table 1 shows the mean values and standard deviations of the VO_2_ max obtained by a graded exercise treadmill test in the lab indicated as VO_2_max (lab); the VO_2_max obtained by substituting body fat percentage value obtained by Jackson and Pollock’s equation(JP eqn) for estimation of body fat through skinfolds indicated as VO_2_ max(J-JP); the Vo_2_ max obtained by substituting body fat percentage value obtained from Durnin and Womerslay’s equation(DWs eqn) [17] for estimation of body fat percentage indicated as VO_2_ max (J-DW); and theVO_2_max obtained by substituting body fat percentage obtained from Sandhu et al’s [18] body fat equation(Ss eqn) for the estimation of fat mass indicated as VO_2_ max (J-S). This table indicates that VO_2_ max (J-S) gives a mean value (36.05 + _6.04 ml/kg/min) which is closest to the measured VO_2_max (mean value of 33.41 + _14.39 ml/kg/min) as compared to VO_2_max (J-JP) and Vo_2_max (J-DW) which clearly overestimate the VO_2_ max in our subjects. ANOVA values indicated in Table 2 further highlight the fact that the differences between the values for VO_2_ max obtained from the 3 equations and measured lab value are statistically significant. Post hoc analysis using Scheffe’s test in Table 3 shows that the difference between VO_2_max (lab) and VO_2_max (J-S) is not statistically significant (p = 0.226) but the differences between VO_2_max (lab) and VO_2_ max(J-JP) and VO_2_max(J-DW) are statistically significant(p < 0.0001). The application of Inter class correlation matrix (Table 4) further indicates that Vo_2_ max (J-S) is slightly more correlated to the measured VO_2_ max than the others. Table 2 also indicates the analysis of variance for percentage body fat. The “F” value 191.103 is highly significant. The application of post hoc Scheffe’s test indicates that all 3 body fat percentages were significantly different (p < 0.05).

**Table 1:** **VO**_**2 **_**max values obtained by the substitution of body fat values obtained by different formulae**

	**Mean ± SD ml/kg/min**
**VO**_**2 **_**max (Lab)**	**33.41 ± 14.39**
**1. VO**_**2 **_**max (J-JP)**	**43.25 ± 7.81**
**2. VO**_**2 **_**max (J-DW)**	**40.47 ± 8.79**
**3. VO**_**2 **_**max (J-S)**	**36.05 ± 6.04**

**Table 2:** **Analysis of Variances (ANOVA) for VO**_**2**_**max and percentage Body fat from Jackson Pollock, Durnin Womersley and Sandhu**

**1. VO**_**2 **_**Max**	**Sum of squares**	**df**	**Mean square**	**F**	**Sig.**
Between Groups	6977.761	3	2325.920	24.343	.000*
Within Groups	45480.583	476	95.547		
Total	52458.345	479			
**2. percentage Body Fat**	Sum of Squares	df	Mean Square	F	Sig.
Between Groups	8237.445	2	4118.722	191.103	.000*
Within Groups	3814.765	177	21.552		
Total	12052.210	179			

*The mean difference is significant at the 0.05 level.

**Table 3:** Post Hoc Test Scheffe’s for VO2max(lab),VO2max (J-JP),VO2max (J-DW) and VO2max (J-S)

	**Variables**	**Sig.**	**95percentage confidence interval**	
			**Lower Bound**	**Upper Bound**
VO_2_max (lab)	VO_2_max (J-JP)	.000*	−13.3742	−6.2935
	VO_2_max (J-DW)	.000*	−10.6037	−3.5230
	VO_2 _max (J-S)	.226	−6.1785	.9022
VO_2_max (J-JP)				
	VO_2_max (J-DW)	.187	-.7698	6.3109
	VO_2 _max (J-S)	.000*.	3.6554	10.7361
VO_2_max (J-DW)				
	VO_2 _max (J-S)	.007*	.8849	7.9656

*. The mean difference is significant at the 0.05 level.

**Table 4:** Inter class correlation between VO2max(lab),VO2max (J-JP),VO2max (J-DW) and VO2max (J-S)

	**Vo2max (lab)**	**VO2(J-JP)**	**VO2(J-DW)**	**VO2(J-S)**
Vo2max	1.000	.807	.814	.817

## Discussion

In this study we measured the VO_2_max by means of a GXT in college aged participants in India. This study was conducted to check the validity of Jackson et al’s [6] VO_2_max prediction equation using percentage body fat as a variable. An earlier study [21] has indicated that obese individuals have a reduced (VO_2_max) relative to body weight thus indicating that percentage body fat has a significant effect on the aerobic capacity (VO_2_max) of an individual. Secondary to this is the fact that body composition varies with ethnicity [7]. Recent evidence in the field of body composition has indicated that racial differences may exist in body composition. Specifically researchers [28] have indicated that Asians had more subcutaneous fat than whites and had different fat distributions from whites. Asians had more upper-body subcutaneous fat than whites. Researchers [28] have also indicated that BMI for given weight under predicts the percentage body fat in Asian Indians. A study **[**[29] has indicated that for the same BMI, percentage BF for Pacific Island men was 4percentage points lower and for Asian Indian men was 7–8percentage points higher compared to Europeans. Compared to European men for the same percentage BF, BMI was 2–3 units higher for Pacific Island and 3–6 units lower for Asian Indians. Asian Indians have more abdominal fat deposition than their European and Pacific Island counterparts. Considering this evidence we believed that prediction equations for the estimation of VO_2_max would be inaccurate and substituting percentage body fat values obtained from formulae developed specifically for Asian Indians would be better correlated to lab measured values of VO_2_max.

Our results indicated that our Asian Indian subjects indeed did have lower VO_2_max values as compared to age matched subjects from Caucasian populations indicating a lower level of fitness. Specifically we recorded a mean value of 33.41 ± 14.39 ml/kg/min which is much lower than those recorded by other investigators in age matched subjects (Heil *et al [*[5]. :45.83 ± 6.57 ml/kg/min and Bradshaw *et al.*[22] :45.17 + −7.59 ml/kg/min). We believe the lower levels could be because of two reasons. Firstly as reported by previous investigations our subjects did indeed have greater amounts of fat mass for a given weight as compared to other ethnicities which would indeed hamper fitness and not contribute to metabolic activity. Secondly is observation that in general South Asians have lower levels of physical fitness than their European counterparts [30,31]. Reasons for this have been suggested to a more restricted range of sporting and leisure activities than Europeans [30].

We also found that Jackson et als’ [6] predictive equation overestimated the VO_2_max compared to the measured value irrespective of which of the three studied equations was used to estimate the fat percentage of subjects. However of the three studied equations we found that substituting fat percentage values obtained from Sandhu et al’s [18] fat mass equation yielded a VO_2_max value that was closer to the measured value as compared to the other two equations. This better correlation observed could be due to the fact that the indigenously developed body fat mass equation generated by Sandhu *et al.,* 2010 **[**[18] used actual values of body fat measured using the “gold standard” under water weighing method in its generation and the fact that it was developed on Asian Indian subjects increased its accuracy in this population.

Earlier researchers have proved that Durnin- Womerslay’s **[**[17] equation, which was derived using under-water weighing method, over estimate body fat and percentage body fat in populations of developing countries [32]yet it is widely used in many epidemiological studies. Similarly Jackson and Pollock’s [6] equation also developed in Caucasian populations is also used worldwide for the estimation of body fat percentage.

Our study has many implications. Despite the fact that we had delimited our study to the investigation of only one predictive equation, our study suggests that prediction equations may not be very accurate when used in populations other than those on whom they were generated. Hence they need to be used with caution in other ethnic populations. Though this study indicates that when fat percentage is determined, Sandhu et al's [18] equation is used, the value for VO_2_ max is slightly more reliable than the other two. However, further study regarding its validity when it is used in prediction equation is warranted. Therefore, validation of Sandhu et al’s [18] equation and cross validation of Durnin-Womerslay’s [17] body fat equation is warranted.

## Conclusions

Our results suggest caution in the use of Jackson et al’s [6] equation in Indian population given the observation that it tends to over predict the VO_2_max in Asian Indian subjects. Similarly the validity of equations for the measurement of percentage body fat is also questionable when used in this population. Thus our study supports the development and validation of population specific prediction equations for the estimation of both body fat and VO_2_max.

## Competing interests

The authors declare that they have no competing interests.

## Authors’ contributions

SS conceived and designed the study, interpreted the data, drafted and revised the manuscript. BT carried out the data collection, statistical analysis and interpretation and drafted the manuscript. JS participated in its design, coordination and revised it critically for important intellectual content. All authors read and approved the final manuscript.
